# ENHANCING PALLIATIVE CARE THROUGH THE INTEGRATION OF THE SOCIAL CONVOY: A USER-CENTERED DESIGN APPROACH

**DOI:** 10.1093/geroni/igae098.2070

**Published:** 2024-12-31

**Authors:** Lyndsay DeGroot

**Affiliations:** University of Colorado Anschutz Medical Campus School of Medicine, Aurora, Colorado, United States

## Abstract

Digital health offers considerable potential for enhancing palliative care but often neglects to integrate the patient’s caregiving “social convoy”, i.e. partners, family, and friends. The Social Convoy Palliative Care (Convoy-Pal) mobile intervention was developed using a user-centered design approach involving interprofessional healthcare providers, older adults with serious illness, and their social convoy (N=81). Convoy-Pal, a 12-week intervention, provides a tablet, smartwatch, and options for mobile phone access, with shared access among participants and their convoy. It facilitates assessment, symptom tracking, and provision of palliative care resources based on user responses. Initial usability feedback among 26 participants (16 older adults/10 social convoys) was favorable, particularly regarding the information provided (User Mobile Application Rating Scale-uMARS: 4.22±.8/5). Based on feedback, final iterations were made to improve goal setting, monitoring tools, and convoy assessment. We then conducted a single-site waitlist randomized control trial among N=46 (31 older adults/14 social convoys) to pilot-test the feasibility of delivering Convoy-Pal. Participants were 76.3 years of age, and social convoys 71.6, primarily White, but not all had access to a mobile device/computer. Recruitment of multiple caregivers was challenging; when enrolled the study maintained a 67.4% retention rate with 4.6% missing subscales on surveys. Intervention participants reported improvements in social functioning (SF-36: 64.6±25.8 vs. 73.2±31.3) and social convoys reported increased perceptions of positive aspects of caregiving (29.5±5.28 vs. 35.0±5.35). Usability scores for information provided were again well-rated (uMARS: 3.96±.57/5). This research underscores the importance of incorporating diverse key informant perspectives to develop meaningful and relevant digital palliative care interventions.

